# Future Thinking Priming Especially Effective at Modifying Delay Discounting Rates among Cigarette Smokers

**DOI:** 10.3390/ijerph18168717

**Published:** 2021-08-18

**Authors:** Alina Shevorykin, Warren K. Bickel, Ellen Carl, Christine E. Sheffer

**Affiliations:** 1 Department of Health Behavior, Roswell Park Comprehensive Cancer Center, Buffalo, NY 14203, USA; Ellen.Carl@RoswellPark.org (E.C.); Christine.Sheffer@RoswellPark.org (C.E.S.); 2 Virginia Tech, The Fralin Biomedical Research Institute at VTC, Roanoke, VA 24016, USA; wkbickel@vtc.vt.edu

**Keywords:** tobacco, impulsivity, priming, addiction, rate dependent, delayed discounting

## Abstract

Background: Tobacco use remains one of the world’s greatest preventable causes of death and disease. While most smokers want to quit, few are successful, highlighting a need for novel therapeutic approaches to support cessation efforts. Lower delay discounting (DD) rates are associated with increased smoking cessation success. Future thinking priming (FTP) reliably reduces DD rates in large populations. Smokers consistently discount more than nonsmokers, and evidence suggests that changes in DD rates are rate dependent. This study examined whether smoking status moderated the effect of FTP on DD rates and, if so, if the moderation effect could be attributed to differences in baseline rates of DD. Methods: Moderation analysis was conducted to determine whether the effect of FTP, versus neutral priming (NP), on DD differed among smokers and nonsmokers. Results: Smoking status moderated the effect of condition (FTP vs. NP) on post-intervention DD scores (b = −0.2919, *p* = 0.0124) and DD change scores (b = −0.2975, *p* = 0.0130). There was no evidence of rate dependence effects in the current sample. Conclusions: FTP had a greater effect on decreasing DD rates among smokers than nonsmokers. FTP is effective and simple to administer, which makes it a promising therapeutic approach for aiding smoking cessation.

## 1. Introduction

“The future interests me—I’m going to spend the rest of my life there.”—Mark Twain.

Despite the success of tobacco control efforts, tobacco use remains one of the most significant preventable causes of death and disease in the world today [1]. Annually, cigarette smoking alone causes more than 8 million deaths worldwide, over 600,000 of whom die from secondhand smoke [2,3,4,5,6,7,8]. In the US, smoking cigarettes causes nearly half a million deaths annually and over 30% of all cancer deaths [9,10]. In the US, most individuals who smoke express the desire to quit, and over half make at least one quit attempt nearly every year, but within 6–12 months, over 90% reverse this decision despite significant effort [11]. This pernicious conundrum remains one of the most significant public health challenges today, calling for innovative therapeutic targets and novel approaches to support efforts to quit smoking [10].

Smoking cigarettes is a highly reinforcing experience for many individuals, and achieving long-term abstinence from smoking is a process whereby individuals must repeatedly choose options other than the immediate reinforcing experience of smoking. While the biological rewards (dopamine release) of cigarette smoking are not as significant as those of other drugs, the fact that smoking is so repetitive, and so often performed in conjunction with other activities, increases the association of those rewards with many activities engaged in on a daily basis, which both enhances the pleasure and the motivation derived from the activities and the reinforcing nature of smoking [12,13]. Delay discounting (DD) describes the degree to which the reinforcer’s subjective value declines as the time to receipt increases. Most humans and many animals prefer immediate reinforcement but are willing to wait for a period of time for rewards that are perceived to be of more value [14,15,16,17]. Individuals who smoke demonstrate higher DD rates than individuals who do not, suggesting that, for smokers, reinforcers lose their value quickly when received outside the temporal window for which they are valued [2,3,6,7,8,18,19,20,21,22,23]. Good health, long life, and prudent far-sighted health-related decision-making are all temporally distant reinforcers. Among smokers, lower DD rates predict greater success at achieving and maintaining abstinence from smoking [24,25,26,27,28,29]. DD rate is also modifiable [2,6,26,27,30,31,32,33,34], and thus has become a novel therapeutic target in the treatment of tobacco dependence and other unhealthy behaviors.

Methods to decrease DD rates include framing techniques intended to alter the temporal window in which decisions are made [35]. Episodic future thinking is a framing technique that shows promise for reducing DD rates concurrently with other health behaviors, including cigarette smoking [8,36,37,38,39,40]. Future thinking priming (FTP) is another framing technique administered remotely that reliably reduces DD rates in large populations [41,42]. Framing techniques have been shown to impact the brain via the activation of neural networks involved in decision-making and prospective thinking, resulting in reductions in DD [43,44,45,46].

The effect of FTP on DD rates has been replicated in two studies and with multiple measures of DD with samples that include smokers and nonsmokers [41,42]. However, smokers consistently discount more than nonsmokers [47]. Therefore, understanding whether and how FTP specifically impacts DD rates among individuals who smoke is critical, and should be conducted prior to examining the therapeutic potential of FTP for cigarette smoking. For instance, the higher DD rates found among smokers might be less modifiable given that they appear to be so strongly associated with smoking status. On the other hand, evidence suggests that changes in DD rates are rate dependent [48,49,50,51,52], which suggests that FTP might have a greater impact on smokers, for whom there is more room for change in DD rates.

This study examined whether smoking status moderated the effect of FTP on DD rates and then examined if any differences found could be attributed to rate dependency. This is a secondary analysis of a randomized controlled trial in which FTP significantly decreased DD rates in a large sample of participants [42]. Given the evidence for rate dependence, we hypothesized that FTP would have a greater effect on individuals who smoke than those who do not smoke, and that this would be due to a rate-dependent difference in baseline measures of DD.

## 2. Materials and Methods

### 2.1. Participants

Adult Amazon Mechanical Turk (MTurk) workers (age ≥ 18 years) who spoke English and resided in North America were eligible to participate. The study was approved by the Institutional Review Boards of City University of New York (#680011-1) and Roswell Park Comprehensive Cancer Center (#BDR082917). Informed consent was obtained from all participants. Data was collected in 2016. MTurk is an online worker platform. MTurk research participants provided responses comparable to those of laboratory participants [53,54,55,56].

### 2.2. Study Design and Procedures

In this study, we conceptualized smoking status as a moderator and conducted a moderation analysis to determine whether the relation between the conditions (FTP, NP) and the dependent variable (DD) was moderated by smoking status.

This is a secondary data analysis of a study that compared participants randomized to FTP or neutral priming (NP) in a pre/post-test control group design [42]. Participants (n = 1532) were enrolled and completed baseline measures. Two weeks later, they were randomized, completed the FTP or NP tasks as assigned, and administered the post-intervention assessments.

### 2.3. The Future Thinking Priming and Neutral Tasks

The FTP stimuli consisted of 10 future-oriented words and phrases: “future”, “self-discipline”, “willpower”, “self-control”, “long-term”, “save”, “planned”, and “investment”. NP stimuli consisted of 10 words and phrases that reflected a neutral focus and were shown to have no effect on DD (e.g., “pale”, “informative”, “dispassionate”, “formal”, etc.). Participants were first instructed to write 10 different sentences describing themselves and incorporating at least one of the words provided. After submitting the sentences, participants were instructed to write a short paragraph (not exceeding 250 words) about themselves, incorporating all the words/phrases provided.

### 2.4. Measures

Standard sociodemographic measures were collected at baseline, including sex, age, race, ethnicity, education, and income. Additionally, tobacco use (number of cigarettes smoked per day) and alcohol use (number of alcoholic drinks per week) were collected. Because priming effects are influenced by social self-monitoring [57], we administered the self-monitoring scale. High social self-monitors may initially respond to priming stimuli in a manner consistent with the prime, but they are less likely to maintain primed behaviors because they tend to shift behaviors to match social expectations [57]. Time perspective (future-oriented or not) was assessed with the time perspective questionnaire [58]. Perceived social status (SSS) was measured by the MacArthur Scale of Subjective Social Status [59].

DD was the primary outcome measure. DD of USD 100 was assessed by the 5-Trial adjusting delay discounting task [60]. The 5-Trial task is an interactive instrument that automatically adjusts to respondents’ choices to produce a result after a maximum of five trials [60]. Respondents were asked on the first trial whether they would prefer USD 50 now or USD 100 in three weeks. If the immediate option is selected, then the second trial shortens the delay to one day (USD 50 now or USD 100 in one day). If the delayed option is selected on the first trial, then the second trial lengthens the delay (i.e., USD 50 now or USD 100 in two years). Delays on all subsequent trials are adjusted based on responses from the preceding trial. The 5-trial task output was expressed as the natural logarithm of k in Mazur’s hyperbolic discounting model, with k increasing as the preference for smaller sooner rewards increases [17]. Lower k values mean that individuals are more willing to wait for a larger reward.

### 2.5. Data Analysis

The analysis was carried out using IBM SPSS, Version 23 (IBM Corp., Armonk, NY, USA). Descriptive analyses were conducted to characterize the sample. One-way analysis of variance (ANOVA) and χ^2^ analysis were used to examine characteristic differences between smokers and nonsmokers. Statistically significant differences between the smokers and nonsmokers were included in the final models to control for baseline differences between the groups.

Moderation analysis was conducted using Hayes PROCESS v3.5.3 with bootstrapping at 1000 samples to examine potential moderation of smoking status on the effects of FTP on DD. We used two methods of conceptualizing the outcome: (1) DD post-intervention, and (2) change in DD (change = post interventions − baseline). Each outcome was analyzed in a separate model.

In the two models, condition (FTP vs. NP) was entered as a fixed variable, smoking status (smoker vs. nonsmoker) was entered as the moderator, and partner status, income, education, alcoholic drinks, and SSS were entered as covariates to control for baseline differences between smokers and nonsmokers. In the first model, baseline DD rate was also entered to be consistent with Blomqvist’s method of controlling for the baseline measure of interest in the regression analyses [61]. Smoking status was considered a significant moderator if the interaction between smoking status and condition was significant for one or both smoking status categories. If the overall interaction between condition and smoking status was below *p* < 0.10, following the guidance from Hayes, further probing was performed to measure the effects of this interaction on different levels of the moderator [62].

The Oldham correlation method was used to assess rate dependence overall and in smokers versus nonsmokers, using the following equation where *x* is the baseline measure, *y* is the post-intervention measure, *s^2^_x_* is baseline variance, *s^2^_y_* is post-test variance, and *r_xy_* is the correlation between baseline and post-intervention:

$$Oldham\;Corr\;\left(\left(x-y\right),\;\frac{\left(x+y\right)}{2}\right)=\frac{s_{x}^{2}-s_{y}^{2}}{\sqrt{{\left(s_{x}^{2}+s_{y}^{2}\right)}^{2}-4r_{xy}^{2}s_{x}^{2}s_{y}^{2}}}$$

The Oldham correlation method was chosen because it was used to successfully identify rate dependence in DD in other studies and because it removes mathematical coupling, regression to the mean, and mathematical bias [51,53,63]. If the Oldham correlation is above 0.3, the comparison is considered rate dependent [50,51,53,64].

## 3. Results

### 3.1. Participants

Participants’ (n = 1532) mean age was 35.7 years (SD 11.3); 54.8% were female; 82.5% were White; and about half were partnered (58.7%). Two-thirds attended at least some college (65.9%). Annual household incomes ranged from <USD 10,000 (5.1%) to >USD 99,000 (14.3%). The mean score for self-monitoring was intermediate (M = 10.7, SD = 4.7). In the parent study, no characteristic differences among participants randomized to the FTP and NP conditions were found with the exception of other tobacco use (FTP 14.6% versus NP 10.7%; χ^2^ = 5.21, *p* = 0.02) [42].

About one-fifth (22%; n = 333) of participants reported smoking >0 cigarettes per day and were considered smokers. Nonsmokers were more likely to be partnered than smokers (61% vs. 52%); nonsmokers had significantly more education, higher income, and higher SSS than smokers. Smokers reported significantly more alcoholic drinks per week than nonsmokers (5.13 vs. 2.73). See Table 1 for details.

### 3.2. Moderation Effects

**Figure 1:**
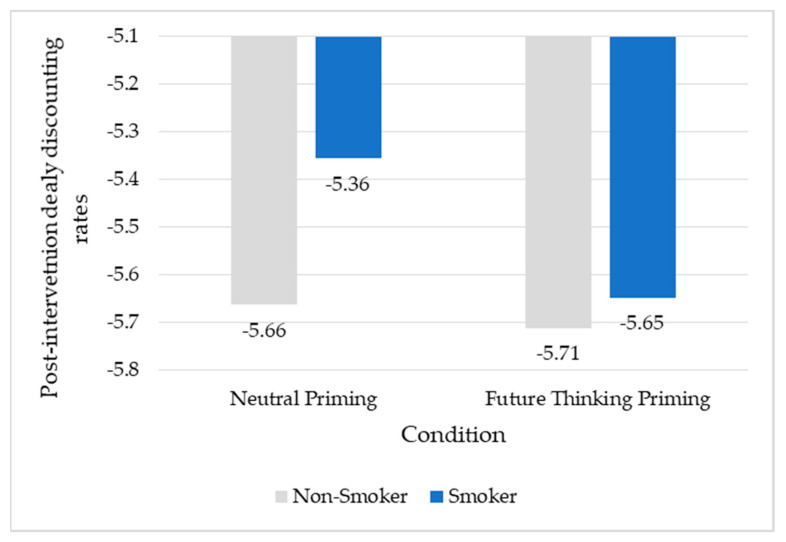
Future thinking priming appears to have a greater effect on smokers than nonsmokers.

*Delay discounting post-intervention*. The overall model for the moderation effect of smoking status on post-test DD rate was significant (R^2^ = 0.6690, F = 341.758, *p* < 0.001). The interaction between condition and smoking status was less than 0.10 (b = −0.2437, *p* = 0.0651). Probing revealed that this effect was significant for smokers (b = −0.2919, *p* = 0.0124). See Table 2 and Figure 1.

**Figure 2:**
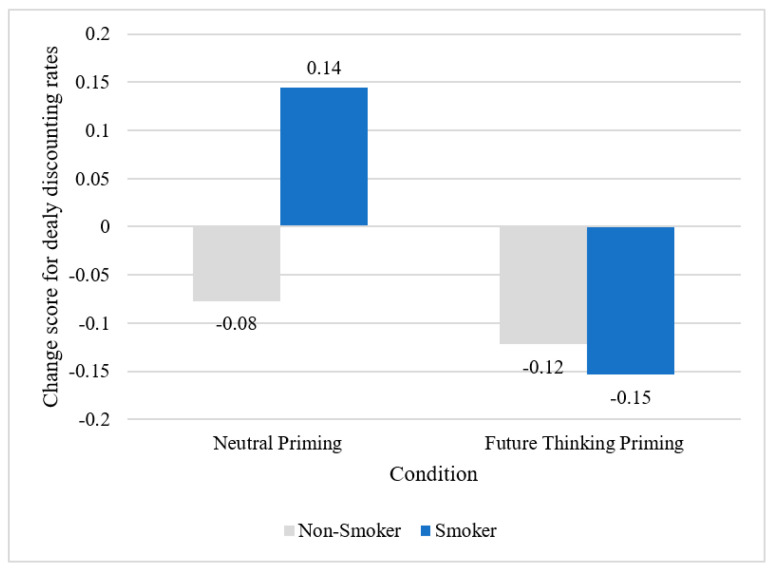
Future thinking priming produced larger change in delayed discounting rate than neutral priming among smokers.

*Change in delay discounting.* The overall model for the moderation effect of smoking status on DD change score was not significant (R^2^ = 0.0070, F = 1.3334, *p* = 0.2223). The interaction between condition and smoking status was less than 0.10 (b = −0.2525, *p* = 0.0620). Probing revealed this effect was significant for smokers (b = −0.2975, *p* = 0.0130) (see Table 3 and Figure 2).

### 3.3. Rate-Dependence Effects

No evidence of rate dependence was found. All Oldham correlations were below the 0.3 threshold. The overall DD outcomes showed an Oldham correlation of −0.113; DD among smokers showed an Oldham correlation of −0.010; DD among nonsmokers showed an Oldham correlation of −0.140.

## 4. Discussion

Our findings indicate that a one-time remote administration of FTP decreased DD among individuals who smoked cigarettes. The FTP task had a greater effect on DD rates among individuals who smoked cigarettes than among those who did not. This suggests that FTP can modify DD rates among smokers, which enhances its therapeutic potential as a novel intervention to reduce DD and concurrently reduce cigarette consumption among smokers, similar to episodic future thinking. Our robust approach examined the moderating effects of smoking status on the effects of FTP on DD rates, using two different methods to account for baseline DD rates. Smoking status had a significant moderating effect in both models, which reinforces the reliability and validity of the findings.

FTP shows promise for development into a novel intervention to support smoking cessation. More research is needed to determine if FTP can decrease cigarette consumption as it decreases DD rates and to determine whether repeated exposure provides cumulative effects. Determination of optimal exposure patterns to support short- and long-term cessation is needed prior to efficacy testing.

If found to support smoking cessation, FTP has the potential to be disseminated widely with minimal infrastructure, which bodes well for potential reach. The FTP task was developed and tested via remote administration using an easily accessible survey program such as Qualtrics or RedCap. Individuals’ engagement in the FTP task is practical and relatively undemanding. Individuals can engage in the task via a link sent by text or email and complete the task on a computer or mobile device.

Given the role of temporal distance in the subjective value of reinforcers, temporal orientation is increasingly being recognized as an important factor in attaining and maintaining abstinence from smoking. Efforts to develop novel interventions that target DD are gaining traction. These findings support previous research focused on DD as a therapeutic target in the treatment of tobacco use [2,3,6,7,8] and represent the next step in the examination of FTP as an effective intervention.

We did not find rate-dependence effects, which might be indicative of the characteristics of the sample. Rate dependence is understudied, but we believe the process of calculating rate dependence is accessible, and future studies should continue to investigate rate dependence as part of the standard procedure [48,49,51,52,65].

This study has several important strengths. First, the current sample was relatively large and representative, which increases the generalizability of findings. Second, results were replicated in two different models, which reduces the likelihood of potential model error or bias. Third, the rigorous design of the parent study helped ensure participant understanding of instructions and accuracy of measurements. Fourth, the current study addresses rate-dependence effects, which has recently been recognized as a methodological standard in research on treatment effectiveness [49,51,52,65]. Finally, the fact that the current study is a secondary data analysis helps increase the impact of the parent study and expands the research knowledge by investigating additional hypotheses cost-effectively and efficiently, as well as helping identify differences in treatment among subgroups of the parent study, which in turn can inform tailoring of group-specific treatments [64,66].

This study has several limitations as well. The moderating effect of smoking status on the effects of FTP on DD rates might be explained by an unknown third factor, for example, having a higher income bracket might contribute to more future thinking orientation in general. Although relatively large, the sample sizes were different for nonsmokers and smokers. This might have contributed to a lack of significance in the overall interactions. There was no conditional effect for nonsmokers and they were a much larger group. Therefore, their weight in the standard error was greater. Finally, smoking status is a self-selected characteristic which ultimately may have resulted in some differences in psychosocial measures, even though these were controlled statistically in all models.

## 5. Conclusions

The current study showed that one-time administration of FTP had a greater effect on decreasing DD rates among smokers than nonsmokers. The FTP is a simple task accessible remotely on a wide variety of devices. The FTP task has potential to be developed as a therapeutic support for smoking cessation.

## Figures and Tables

**Table 1:** Participant characteristics.

Characteristic/Variable		Range, Level, Category	Mean (SD) or Percent (N)		χ^2^ or F
			Nonsmokers (n = 1199)	Smokers (n = 333)	
Sociodemographic	Sex	Female	55.96 (671)	50.75 (169)	χ^2^ = 2.859, *p* = 0.091
	Age	18–74	35.40 (11.27)	36.74 (11.201)	F = 3.714, *p* = 0.054
	Race	White	81.81 (981)	84.98 (284)	χ^2^ = 3.361, *p* = 0.339
		Black	6.26 (75)	6.31 (21)	
		Asian	6.00 (72)	3.60 (12)	
		Other	5.92 (71)	5.11 (17)	
	Hispanic	Yes	6.42 (77)	6.91 (23)	χ^2^ = 3.361, *p* = 0.751
	Partner status *	Partnered	60.71 (728)	51.65 (172)	χ^2^ = 8.838, *p* = 0.003
	Education in years *	1–28	15.81 (2.686)	14.62 (2.611)	F = 51.561, *p* < 0.001
	Annual household income *	<USD 24,999	17.10 (205)	28.53 (95)	χ^2^ = 52.556, *p* < 0.001
		USD 25,000–USD 49,999	28.86 (346)	35.74 (119)	
		USD 50,000–USD 74,999	22.85 (274)	22.52 (75)	
		USD 75,000–USD 99,999	15.01 (180)	5.71 (19)	
		>USD 100,000	16.18 (194)	7.51 (25)	
Alcohol use	Number of drinks per week *	0–70	2.73 (5.155)	5.13 (8.049)	F = 43.077, *p* < 0.001
Psychosocial	Self-monitoring scale	0–25	10.59 (4.598)	11.01 (4.960)	F = 2.157, *p* < 0.142
	Time perspective	Future oriented	66.31 (795)	63.96 (213)	χ^2^ = 0.635, *p* = 0.426
	SSS *	0–10	4.91 (1.839)	4.17 (1.838)	F = 41.979, *p* < 0.001

*p < 0.05. SSS is perceived social status.

**Table 2:** Moderating effect of smoking status on post-intervention delay discounting rate.

Outcome Measure	Factors/Variables		B	SE	t	*p*	95% CI	
							Lower	Upper
Post-intervention delay discounting rate	Interaction + effects	nonsmokers	−0.0482	0.0616	−0.7836	0.4334	−0.1690	0.0725
		smokers	−0.2919	0.1168	−2.499	0.0124	−0.5209	−0.0629
	Constant		−0.5631	0.1964	−2.8672	0.0042	−0.9483	−0.1779
	Condition		−0.0486	0.0616	−0.7901	0.4296	−0.1694	0.0721
	Smoking status		0.3077	0.0968	3.1777	0.0015	0.1177	0.4976
	Baseline delay discounting rate		0.8579	0.0163	52.6498	0.0000	0.8259	0.8898
	Partnered status		0.0095	0.0592	0.1598	0.8731	−0.1066	0.1256
	Education		−0.0192	0.0106	−1.8075	0.0709	−0.0401	0.0016
	Income		−0.0076	0.0183	−0.4139	0.6790	−0.0436	0.0284
	Alcohol (number of drinks per week)		−0.0057	0.0046	−1.2234	0.2214	−0.0147	0.0034
	Perceived social status (SSS)		0.0039	0.0183	0.2125	0.8317	−0.0320	0.0398

+ Interaction is the conditional effects of the condition type (FTP vs. NP) based on the moderator variable (nonsmokers vs. smokers).

**Table 3:** Moderating effect of smoking status on change in delay discounting score.

Outcome Measure	Factors/Variables		b	SE	t	*P*	95% CI	
							Lower	Upper
The difference between post-intervention and baseline delayed discounting rate	Interaction + effects	nonsmokers	−0.0450	0.0631	−0.7134	0.4757	−0.1687	0.0787
		smokers	−0.2975	0.1196	−2.4872	0.0130	−0.5321	−0.0629
	Constant		0.0004	0.1900	0.0020	0.9984	−0.3723	0.3730
	Condition		−0.0450	0.0631	−0.7134	0.4757	−0.1687	0.0787
	Smoking status		0.2211	0.0987	2.2410	0.0252	0.0276	0.4146
	Partner		0.0307	0.0606	0.5060	0.6129	−0.0882	0.1495
	Education		−0.0097	0.0108	−0.8933	0.3718	−0.0309	0.0116
	Income		0.0032	0.0188	0.1729	0.8628	−0.0335	0.0400
	Alcohol (number of drinks per week)		−0.0057	0.0047	−1.2065	0.2278	−0.0150	0.0036
	Perceived social status (SSS)		0.0055	0.0188	0.2911	0.7710	−0.0313	0.0423

+ Interaction is the conditional effects of the condition type (FTP vs. NP) based on the moderator variable levels (nonsmokers vs. smokers).

## Data Availability

No new data were created or analyzed in this study. Data sharing is not applicable to this article.
